# Does knowledge brokering improve the quality of rapid review proposals? A before and after study

**DOI:** 10.1186/s13643-017-0411-0

**Published:** 2017-01-28

**Authors:** Gabriel Moore, Sally Redman, Catherine D’Este, Steve Makkar, Tari Turner

**Affiliations:** 1 School of Public Health, Sydney Medical School, The University of Sydney, Edward Ford Building (A27), Sydney, NSW 2006 Australia; 2 The Sax Institute, Level 13, Building 10, 235 Jones Street, Ultimo, NSW 2007 Australia; 3 National Centre for Epidemiology and Population Health (NCEPH), Research School of Population Health, The Australian National University, 62 Mills Road, Acton, ACT 0200 Australia; 4 School of Public Health and Preventive Medicine, Monash University, Level 1, 549 St Kilda Road, Melbourne, VIC 3004 Australia

**Keywords:** Knowledge brokering, Rapid reviews, Commissioned rapid reviews, Review literature as topic, Knowledge synthesis, Evidence summaries, Research utilisation, Policy-relevant

## Abstract

**Background:**

Rapid reviews are increasingly being used to help policy makers access research in short time frames. A clear articulation of the review’s purpose, questions, scope, methods and reporting format is thought to improve the quality and generalisability of review findings. The aim of the study is to explore the effectiveness of knowledge brokering in improving the perceived clarity of rapid review proposals from the perspective of potential reviewers.

To conduct the study, we drew on the Evidence Check program, where policy makers draft a review proposal (a *pre* knowledge brokering proposal) and have a 1-hour session with a knowledge broker, who re-drafts the proposal based on the discussion (a *post* knowledge brokering proposal).

**Methods:**

We asked 30 reviewers who had previously undertaken Evidence Check reviews to examine the quality of 60 *pre* and 60 *post* knowledge brokering proposals. Reviewers were blind to whether the review proposals they received were *pre* or *post* knowledge brokering.

Using a six-point Likert scale, reviewers scored six questions examining clarity of information about the review’s purpose, questions, scope, method and format and reviewers’ confidence that they could meet policy makers’ needs. Each reviewer was allocated two *pre *and two *post* knowledge brokering proposals, randomly ordered, from the 60 reviews, ensuring no reviewer received a *pre* and *post* knowledge brokering proposal from the same review.

**Results:**

The results showed that knowledge brokering significantly improved the scores for all six questions addressing the perceived clarity of the review proposal and confidence in meeting policy makers’ needs; with average changes of 0.68 to 1.23 from *pre* to *post* across the six domains.

**Conclusions:**

This study found that knowledge brokering increased the perceived clarity of information provided in Evidence Check rapid review proposals and the confidence of reviewers that they could meet policy makers’ needs. Further research is needed to identify how the knowledge brokering process achieves these improvements and to test the applicability of the findings in other rapid review programs.

**Electronic supplementary material:**

The online version of this article (doi:10.1186/s13643-017-0411-0) contains supplementary material, which is available to authorized users.

## Background

The use of evidence from research in the development of health policy has the potential to inform decision-making and improve health outcomes. However, it is widely recognised that many opportunities to use research are currently missed [1, 2].

Policy makers and program managers have reported barriers and facilitators to using research evidence in policy-making [3, 4]. For example, a recent systematic review found that the most frequently reported barriers to the use of evidence were poor access to good quality, relevant research and a lack of timely research output [5]. Policy makers also point to a number of strategies they think might work to increase their use of research. In particular, they have identified the need to improve access to summaries, reviews and syntheses of research [1, 5, 6].

Rapid reviews of research can improve timely access to relevant research for decision-making. Rapid reviews provide information about what evidence exists, where there are gaps in the evidence, an evaluation of the quality of the evidence and the researchers’ understanding of the implications of this evidence for policy-making [7, 8].

Rapid review programs are increasingly being implemented. For example, in Canada, the Knowledge to Action research program provides rapid reviews of research in Ottawa [9, 10], and the McMaster Health Forum provides rapid response documents on 10- or 30-day time frames [11, 12]. In UK, the Government Social Research Service enables commissioned rapid evidence assessments [13, 14], and the King’s Fund produces rapid evidence reviews [15]. In USA, the Veterans Affairs Evidence-based Synthesis Program [16, 17] and the Agency for Healthcare Research and Quality’s Evidence-based Practice Centers [18] produce syntheses of targeted health care topics of particular importance to policy makers and managers. Furthermore, Cochrane Innovations provides rapid reviews to support evidence-informed decision-making for policy makers and healthcare professionals [19].

There is growing interest in how such reviews might be made as useful as possible [6, 9]. Reviewers need a clear understanding of policy makers’ requirements to provide useful reviews [6, 9, 20]. Early studies point to the importance of a clear articulation of the review’s purpose, questions, scope and methods [9, 21, 22]. These factors have been argued to improve the quality and generalisability of rapid review findings [22].

In contrast to systematic reviews, rapid reviews are frequently commissioned in response to questions arising from immediate policy processes [23]. Review findings are therefore intended to address a particular policy issue, in a specific context and time. If a rapid review is to be useful, understanding how a policy issue arose and the policy maker’s purpose in commissioning the review will be important [21, 24]. The purpose of the review will inform the review questions, scope, methods and format.

The task of translating policy questions into specific questions that can be addressed through a rapid review is surprisingly complex [20]. The review questions must be appropriate both from a policy perspective (reflecting the subject matter and focus of the review) and from a research perspective (be amenable to research processes such as defining search terms and inclusion criteria). A clear scope for a rapid review should ‘set boundaries’ and define key terms [20] so they are consistent with the policy makers’ intent and provide guidance for the reviewers in terms of their search strategy, screening of papers, data extraction and analysis. Ideally, the scope of the review is identified at the outset between the policy agency and the reviewer and outlines inclusion and exclusion criteria, evidence sources and timelines, and the geographic boundaries and languages of the review.

The need for transparent reporting of rapid review methods has been acknowledged [20, 22]. A variety of techniques have been used to make reviews more rapid, including narrowing search strategies, limiting study types, accelerating data extraction and limiting quality assessment. A clear articulation of these methods in rapid reviews is critical in determining the reliability and validity of the findings.

Despite the agreed importance of outlining a detailed scope and methods for rapid reviews [23, 25], to date, there has been only limited investigation of how this might best be done. It has been suggested that tailored and customised formats will increase the extent to which rapid reviews are useful to policy makers [6]. Standardised products may run the risk of reducing the usability of a particular report, as topic areas, review questions and policy contexts vary considerably across and within agencies.

Knowledge brokering has frequently been proposed as a strategy to increase the use of research in policy-making [23, 25]. Knowledge brokers have been described as skilled communicators whose extensive experience in both policy and research enables their unique capacity to work within and across policy and research contexts [25–27]. Knowledge brokering is thought to facilitate access to research and research findings [28], assist in translating research into policy [29, 30], harness the expertise of policy makers and researchers, and sustain their ongoing engagement [31, 32]. Knowledge brokering has been used at the interface between policy and research to clarify information needs, define researchable policy questions, commission syntheses of research and report on their findings [33, 34]. However, little is known about the impact of knowledge brokering in practice. We found only one study which tested the impact of knowledge brokering on the use of research by policy makers and, though there were benefits to organisations with low levels of research receptivity, no effect overall was demonstrated in evidence-informed decision-making [35, 36]. To date, no studies have reported on the effectiveness of knowledge brokering in commissioning rapid reviews.

The Sax Institute’s Evidence Check rapid review program was developed to assist organisations gather the best and most relevant research evidence to inform their policy-making and program development. As part of the program, knowledge brokers work with a policy or program team to help them clarify their policy issue, increase the accuracy of their review questions, clarify the review scope and methods and help determine timelines, budgets and reporting formats [37]. Knowledge brokers are senior researchers with extensive experience working with policy agencies.

In the Evidence Check process, policy makers and program managers complete a draft review proposal before knowledge brokering (a *pre* knowledge brokering proposal), describing their policy or program issue and proposed review questions. After structured and tailored discussion with the policy team, the knowledge broker drafts a synthesis of the discussion which is agreed with the policy team (a *post* knowledge brokering proposal). This *post* knowledge brokering proposal is given to the review authors who will undertake the review, defining its parameters. To date, more than 200 reviews have been commissioned through this process. More information about the Evidence Check process can be found on the Sax Institute website [38].

The aim of the study was to explore whether knowledge brokering, undertaken as part of the Evidence Check program, improved the perceived clarity of the policy agencies’ review proposals from the perspective of potential reviewers, in relation to the purpose of the review, the review questions, the scope and method, and the information to be included in the report; and improved the confidence of reviewers that they could produce a report that would meet policy makers’ needs based on the information contained in the proposal.

## Methods

### Study design

Individuals who had previously undertaken reviews through the Evidence Check program were asked to score *pre* and *post* knowledge brokering proposals in terms of their perceived clarity; previous reviewers were selected as the study participants as we regarded their views as most likely to reflect those of other reviewers in the Evidence Check program, and we refer to them as ‘representative reviewers’. Representative reviewers were blind to whether the proposal was written *pre *or *post* knowledge brokering. Thirty representative reviewers were included in the study, and each received four proposals from 120 in total, from 60 rapid reviews.

### Study sample

All Evidence Check reviews commissioned between 1 January 2006 and 30 June 2013, with both a *pre* knowledge brokering and a *post* knowledge brokering proposal in a standard format were eligible for inclusion in the study. Of the 120 reviews commissioned, 85 met the inclusion criteria. Sixty reviews were randomly selected, giving a total sample of 120 proposals: 60 *pre *knowledge brokering and 60 *post* knowledge brokering. All proposals were presented in a uniform design and format and were randomly assigned a number from 1 to 60. Business information, such as publication and payment arrangements, was removed from the proposals.

Seventy-seven first authors of reviews previously commissioned through the Evidence Check program were invited to participate in the study, as representative of potential reviewers. The first 30 representative reviewers to respond were randomly assigned a unique identification number (numbered 1–30) by a research assistant not otherwise involved in this study, ensuring that the study authors were blinded to the identity of the representative reviewers.

### Allocation of reviews

Each representative reviewer was allocated two *pre* and two *post* knowledge brokering proposals in sequential order so they did not receive *pre* and *post* proposals from the same review, and with the restriction that they did not receive a proposal for a review they had undertaken. For example, representative reviewer 1 was allocated *pre* knowledge brokering proposals from reviews 1 and 2 and *post* knowledge brokering proposals from reviews 3 and 4; representative reviewer 2 was allocated *pre* knowledge brokering proposals from reviews 3 and 4 and *post* knowledge brokering proposals from reviews 5 and 6 and so on. The last representative reviewer (representative reviewer 30) was allocated *pre* knowledge brokering proposals from reviews 59 and 60, and *post* knowledge brokering proposals from reviews 1 and 2. Representative reviewers were blind to whether the proposals they received were *pre* or *post* knowledge brokering. Each representative reviewer scored four proposals, and each proposal was scored once.

Representative reviewers were given 1 month to score the proposals and were sent up to three reminder emails. Each representative reviewer scored each proposal on six questions, using the *perception of proposal questions* described below.

#### The *perception of proposal questions*

Two study authors (GM and TT) developed questions designed to capture the perceptions of representative reviewers about the clarity of the main components of the proposals and about their confidence that they could conduct a review based on the information in the proposals. The questions about clarity were formulated using the standard headings in the *Cochrane Handbook >for Systematic Reviews of Interventions* (http://handbook.cochrane.org/) as a starting point. Respondents were given the following response options using a Likert scale approach. The response options ranged from ‘very unclear/very unconfident’ to ‘very clear/very confident’. We refer to these questions as the *perception of proposal questions *throughout. The *perception of proposal questions* were examined by three individuals not participating in the main study but with the same characteristics as the representative reviewers. Each individual was asked to indicate their preferred version of the scale and was interviewed about the clarity, appropriateness and relevance of the questions as proposed by Holden [39]. The individuals were able to complete all the *perception of proposal questions* and indicated that they found the questions clear and easy to use. Based on the results of the pilot testing, the questionnaire was reduced in length for feasibility, and ease of use and minor amendments were made to the instructions for representative reviewers. The questionnaire was restructured to capture the main content areas only, resulting in five questions about the clarity of information; a sixth question was added about representative reviewers’ confidence, and a seventh optional question allowed them to comment on their experience of scoring the proposals. An example of the *perception of proposal questions* is provided in Additional file 1.

The final *perception of proposal questions* included the following questions: (1) How clear is the proposal about why the policy maker is commissioning the review? (2) How clearly articulated are the questions to be answered in this review? (3) How clearly described is the scope of the review? (4) How clearly described is the method of the review? (5) How clear is the proposal about what should be included in the report? (6) Based on the information provided in the proposal, how confident are you that a researcher in this field will know enough to provide a rapid review of the literature that will meet the policy maker’s needs? (7) Is there anything else you would like to tell us? Respondents were required to answer these questions based on the material in the proposal.

### Data analysis

The data were analysed using Stata version 13. For each question, a mixed model was generated with crossed effects for review and representative reviewer both at level 2 [40, 41]. Specifically,*pre* and *post* proposals (*n* = 120) were nested within both reviews (*n* = 60) and representative reviewers (*n* = 30), but the reviews were not nested within representative reviewers. A *pre/post* variable which was statistically significantly different from 0 indicated that there was a significant change in score from *pre* to *post* knowledge brokering. The analysis used a random effects model, which is valid under the assumptions that data are missing at random, conditional on the covariates included in the model. The reviewer, the proposal and the score were all included in the analysis, to manage any issues associated with missing values.

A sample size of 60 reviews and 30 representative reviewers would allow us to detect a difference in outcomes from *pre* to *post *knowledge brokering of approximately half a standard deviation, with 80% power, a 5% significance level, a *pre-post *score correlation of 0.4 or more and a design effect of 1.5 due to correlation of observations within representative reviewers. We considered this *pre-post* difference to be a clinically important difference in the quality of the proposals.

Illustrative examples were selected by one of the study authors (GM) from the proposals; permission was sought from the relevant agencies for inclusion of the examples where possible, and otherwise, all identifying information was removed.

The dataset supporting the conclusions of this article is included within the article and its Additional file 2.

## Results

Ninety-eight of the 120 documents were returned (82%). Of these, 82 documents were *pre* and *post* from the same review, and 16 were either *pre* or *post* documents only (eight *pre* and eight *post*). Five representative reviewers returned no documents (*n* = 20 missing), and two representative reviewers returned three out of four documents (*n* = 2 missing).

### Quantitative analysis

Table 1 shows the mean, standard deviation and effect size for changes from the *pre* to *post* knowledge brokering scores.

**Table 1:** Mean and standard deviation of *pre* and *post* knowledge brokering scores for each domain

	*Pre* knowledge brokering		*Post* knowledge brokering		Change from *pre* to *post* knowledge brokering			
	Mean	*SD*	Mean	*SD*	Mean^a^	95% confidence limits		*p*
						Lower	Upper	
Clarity about why the review was commissioned	4.67	1.46	5.37	0.95	0.681	0.249	1.114	0.002
Clarity of the review questions	*4.47*	1.39	*5.22*	1.01	0.755	0.282	1.228	0.002
Clarity of the scope	*3.92*	1.41	*5.16*	1.08	1.228	0.816	1.641	<0.001
Clarity of the method	*3.883*	1.32	*4.55*	1.28	0.669	0.308	1.029	<0.001
Clarity of the report inclusions	*4.35*	1.35	*5.37*	0.78	1.026	0.612	1.439	<0.001
Reviewers’ confidence	*3.84*	1.30	*4.86*	1.19	1.018	0.537	1.500	<0.001

^a^Note that the estimate represents the mean change from *pre* to *post* (*post* score–*pre* score), i.e. a positive value indicates that the *post* knowledge brokering score is higher than the *pre* knowledge brokering score

*p* value from Wald test

The mean scores were significantly higher for the *post* knowledge brokering proposals compared to that for *pre* knowledge brokering proposals for all six questions. The mean difference in scores from *pre* to *post* knowledge brokering ranged from 0.68 for question 4 to 1.23 for question 3, after adjusting for review and representative reviewer (see Table 1).

### Description of changes following knowledge brokering

The mean score for perceived clarity about why the review was commissioned changed from 4.67 to 5.37 following knowledge brokering. *Pre* knowledge brokering proposals often lacked the details representative reviewers needed to provide a review which would be relevant and useful. They included statements such as ‘the purpose of the review is to inform the agency’s planning and implementation’ or ‘the review will inform the design of a new model of care’. *Post* knowledge brokering, additional information was provided about how the review would be used and by whom. For example, ‘The review will be used to develop a model of care for improved funding and delivery of [service type] services in hospital and community settings in NSW. The review will also be used in a consultative process to achieve a consensus view among clinicians on the model of care. The audience for this review therefore includes senior policy makers within the [policy agency] and clinicians involved in service planning and care delivery.’

In this study, the mean score for clarity of scope changed from 3.92 to 5.16 following knowledge brokering. The majority of proposals, written* pre* knowledge brokering, provided only limited information such as data sources or years. Examples of scope *pre* knowledge brokering included ‘Reviewers should search academic databases, and the grey literature’ or ‘the target population is adults’. *Post* knowledge brokering, much more detail was provided about the policy makers’ focus of interest. One example of this was ‘The review should focus on changes in [service type] and in [population subgroups] such as children, adolescents, older people, [age ranges specified], those living outside major metropolitan areas, or those from culturally and linguistically diverse backgrounds. To be relevant, the included evidence must provide details of the context in which the change in [service type] was implemented, such as organisational models, funding models, cultural context, political context, health workforce, or governance structures. While evidence from Australian jurisdictions is preferred, evidence from other countries with similarly advanced healthcare systems is applicable (for example, UK, Western Europe, Canada, USA, New Zealand).’

Mean scores in this study about the perceived clarity of the methods changed from 3.88 to 4.55 following knowledge brokering. Most *pre* knowledge brokering proposals in our study provided only general statements such as ‘the reviewer should comment on the quality of the findings’ or ‘the report should include tables of the relevant papers indicating the methods and findings’. *Post* knowledge brokering an example read ‘The review should provide a brief summary of existing reviews of the evidence, including clinical guidelines, with an expert recommendation, and analysis of the applicability of the findings to NSW. The reviewer should include opinion about the quality and strength of the findings and identify the gaps in the evidence, including studies with insufficient data and/or low quality methodology for measuring an effect size. In assessing the quality of the included studies, the reviewer may use existing quality assessment guidelines (such as that issued by the NHMRC), or define a scale (e.g. weak/moderate/strong).’

## Discussion

This study found significant improvements, following knowledge brokering, in the perceived clarity of information provided in Evidence Check rapid review proposals about their purpose, review questions, scope, method, and report format, and in representative reviewers’ confidence that they could meet policy makers’ needs. These differences remained even after controlling for representative reviewer and reviews.

The examples provided demonstrate the ways in which often very loosely defined questions were refined, expanded and clarified to provide much more contextual information and direction for the representative reviewer following discussion with the knowledge broker.

The role of knowledge brokers in our study differs from those of other studies in some important ways. For example, the work of knowledge brokers often focuses on developing the research capacity of policy makers or on supporting agencies to apply research in policies and programs [35]. Knowledge brokering interventions are often multi-faceted and may be sustained for periods of months or years [30]. In contrast, this study demonstrates that knowledge brokering may also be effective in one-off, brief interventions.

**Fig. 1:**
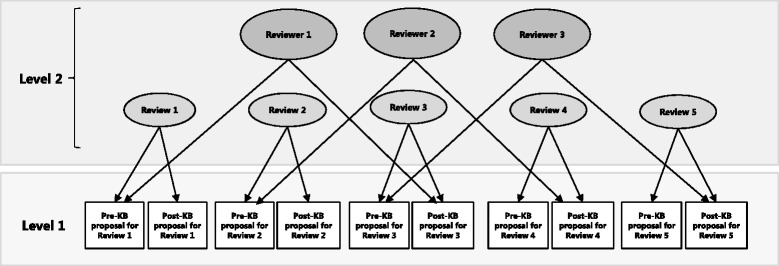
Cross-classified data structure and allocation of documents used in the study. Note that the figure represents the data structure and allocation of documents for the first three reviewers only and shows the multi-level model used in the study

The study design ensured that representative reviewers were randomly assigned documents with the restriction that they could not receive a *pre* and *post* knowledge brokering proposal from the same review and were blinded to the proposals’ *pre* or *post* knowledge brokering status. Representative reviewers participating in the study were authors of Evidence Check rapid reviews and will have evaluated the proposals from the ‘real-world’ perspective of reviewers conducting the reviews. Further, the study had a larger sample size than the previous studies (60 reviews compared to 11 reviews [9]) and used a multi-level analysis (Fig. 1) that adjusted for the crossed effects of review and representative reviewer.

The findings of this study indicate that representative reviewers believe that review proposals are considerably clearer after knowledge brokering. The illustrative text from proposals provide some examples of the major changes to the descriptions of what is required following the knowledge brokering. On average, the cost of the knowledge brokering session is around $1500 (AUD) adding about 5% to the overall cost of reviews produced through Evidence Check. Subjectively, policy agencies often comment very positively about the value provided by the knowledge brokering; as one policy maker commented: ‘The knowledge broker was very good at quickly understanding the policy issues that we were trying to address; she could very quickly grasp that and was then able to help us define our research questions’ [37]. Indeed many first time users of the Evidence Check program report commissioning subsequent reviews because of the knowledge brokering process. Although it is beyond the scope of the current study, additional work using discrete choice experiments is planned to explore the willingness of policy makers to pay for components such as knowledge brokering [42].

This study has several limitations. Evidence Check uses a standardised process that may be different from those of the other rapid review programs, and knowledge brokering may differentially affect those review proposals. While our study controlled for the effect of reviews and representative reviewers, there may be other factors at work that were not identified in our study. We do not know whether reviews commissioned using other methods would have provided less relevant information, nor can we be certain that the reported changes in clarity actually result in more timely, relevant or useful reports for policy makers. We went to considerable lengths to remove any indication of whether the proposal was *pre* or *post* knowledge brokering, and informal comments suggested that it was not obvious to some representative reviewers. Although it remains possible that some representative reviewers were able to guess whether they were reviewing a *pre* or *post* knowledge brokering proposal, it is not clear that this would result in a significant response bias; that is, even if representative reviewers guessed the status of the proposal, they would have little reason for grading *post *proposals more favourably than *pre* proposals. Further, the examples described above clearly demonstrate the kinds of improvements in clarity that were observed after knowledge brokering, suggesting the findings are not artefactual.

This paper examined the perceptions of representative reviewers about the proposals, but we cannot comment on whether the clarity of the proposal related to the quality of the subsequent review. While it would be interesting to examine the impact on the quality of the reviews themselves, in practical terms, it would be extremely difficult because of the many factors that affect reviews in addition to the proposal. For example, the quality of the review will be affected by the skills of the researcher and the amount and quality of primary research available. Similarly, we acknowledge that we are assessing perceptions of clarity; however, the representative reviewers are experienced reviewers who might be seen as providing an accurate window into the likely views of similar reviewers in the future.

Taken together, these findings suggest that this model of knowledge brokering may be an effective strategy for other agencies wishing to commission rapid reviews and for the researchers who will undertake them. Certainly, in our study, proposals written after knowledge brokering were qualitatively different to those written before knowledge brokering. The key concepts underpinning the review had been defined: the focus and intent of the questions was clear and was matched to the user’s context, the scope was narrowed and consistent with the likely available literature, the methods had been determined and the analytical framework was agreed. While some fine-tuning may be required, a researcher considering undertaking the review will have had clear information about its parameters.

## Conclusions

This study found that following knowledge brokering, there was a significant increase in the perceived clarity of information provided in the Evidence Check rapid review proposals about the review’s purpose, review questions, scope, method and report format, and in the confidence of the representative reviewers that they could conduct a review that would meet the policy makers’ needs. Further research is needed to identify how the knowledge brokering process achieves these improvements and to test the applicability of the findings in other rapid review programs.

## Additional files

Additional file 1:
*Perception of proposal questions* for scoring by reviewers. (PDF 72 kb)
Additional file 2: Representative reviewers’ scores. (XLSX 83 kb)
