# Supplementary material for: Does knowledge brokering improve the quality of rapid review proposals? A before and after study
Source: Syst Rev. 2017 Jan 28;6:23. doi: 10.1186/s13643-017-0411-0 (PMC5273818; doi:10.1186/s13643-017-0411-0)
Supplement: Additional file 1: — Perception of proposal questions for scoring by reviewers. (PDF 72 kb) [file 13643_2017_411_MOESM1_ESM.pdf]

QUESTIONS FOR REVIEWER SCORING PROPOSAL UPN 1

This questionnaire relates to proposal UPN: 1 which is provided below.

Clarity of the information provided

1. How clear is the proposal about why the policy maker is commissioning the review?

   *Please **circle or highlight in bold** the one response which most closely reflects your view.*

Not at all clear	Not very clear	Somewhat unclear	Somewhat clear	Clear	Very clear

2. How clearly articulated are the questions to be answered in this review?

   *Please **circle or highlight in bold** the one response which most closely reflects your view.*

Not at all clearly	Not very clearly	Somewhat unclearly	Somewhat clearly	Clearly	Very clearly

3. How clearly described is the scope of the review?

   *Please **circle or highlight in bold** the one response which most closely reflects your view.*

Not at all clearly	Not very clearly	Somewhat unclearly	Somewhat clearly	Clearly	Very clearly

4. How clearly described is the method of the review?

   *Please **circle or highlight in bold** the one response which most closely reflects your view.*

Not at all clearly	Not very clearly	Somewhat unclearly	Somewhat clearly	Clearly	Very clearly

5. How clear is the proposal about what should be included in the reported?

   *Please **circle or highlight in bold** the one response which most closely reflects your view.*

Not at all clear	Not very clear	Somewhat unclear	Somewhat clear	Clear	Very clear

Overall level of confidence

6. Based on the information provided in the proposal, how confident are you that a researcher in this field will know enough to provide a rapid review of the literature that will meet the policy maker's needs?

   *Please **circle or highlight in bold** the one response which most closely reflects your view.*

Not at all confident	Not very confident	Somewhat unconfident	Somewhat confident	Confident	Very confident

7. Is there anything else you would like to tell us?

   Please add comments here.
